# Effects of High-Fat Diet on Intestinal Microbiota in Largemouth Bass (*Micropterus salmoides*) from a Segmental Perspective

**DOI:** 10.3390/ani16142141

**Published:** 2026-07-09

**Authors:** Yuchen Xiao, Han Dong, Man Xu, Jiale Liu, Jingjing Cheng, Chenyi Tang, Liangchun Yan, Ziyi Xu, Jun Jiang, Haifeng Liu

**Affiliations:** 1 Fisheries College, Sichuan Agricultural University, Chengdu 611130, China; 15883815051@163.com (Y.X.);; 2 Translational Chinese Medicine Key Laboratory of Sichuan Province, Chengdu 610041, China; 3 Sichuan Academy of Chinese Medicine Sciences, Chengdu 610041, China; 4 College of Animal Science and Technology, Sichuan Agricultural University, Chengdu 611130, China

**Keywords:** largemouth bass, gut microbiota, high-fat diet, intestinal segments

## Abstract

Intestinal functional zonation and segment-specific microbiota responses to high-fat diet (HFD) are poorly understood in largemouth bass. In this study, a feeding experiment was conducted using 390 largemouth bass with an initial body weight of 10.87 ± 0.05 g. The impacts of HFDs on intestinal histomorphology and microbial communities across four intestinal segments, compared with a normal diet (ND), using 16S rRNA gene sequencing and histological analysis. The intestine was divided into four segments based on morphological features and length. Results showed that HFDs significantly decreased intestinal fold height and induced inflammatory lesions. Microbial genera exhibited distinct spatial distribution: *Klebsiella* was concentrated in segments 1 and 2, *Cetobacterium* in segment 4, and *Acinetobacter* was abundant in most segments. HFDs significantly increased opportunistic, potentially pathogenic taxa, including *Acinetobacter* and *Plesiomonas*, while reducing probiotics such as *Paenibacillus* and *Bacillus*. Segmented analysis detected more differential microbes and revealed potentially pathogenic taxa overlooked by whole-intestine analysis. These findings reveal segment-specific microbial distribution and HFD-induced intestinal dysbiosis in largemouth bass, providing a theoretical basis for intestinal functional zonation and the optimization of healthy aquaculture strategies.

## Simple Summary

High-fat feeds are widely used to speed up fish growth in bass farming, yet we know little about how such feeds damage bass intestines and their resident microbes across different gut regions. This study split largemouth bass intestines into four separate segments and compared fish fed normal and high-fat diets to fill this research gap. The high-fat diet boosted fish growth rates but cut survival rates, damaged intestinal lining and triggered gut inflammation. Different gut segments held distinct microbial communities. The fatty feed multiplied harmful bacteria and suppressed beneficial microbes that protect intestinal health. Analyzing the whole intestine as one single unit missed many critical microbial shifts unique to specific gut sections. Examining gut segments separately reveals complete changes linked to unhealthy diets. These findings offer practical guidance for adjusting fish feed formulas, protecting bass intestinal health and supporting sustainable, loss-reduced aquaculture production.

## 1. Introduction

The gut microbiota plays a crucial role in maintaining host health [1,2] and exhibits extensive potential for diverse applications. For example, *Cetobacterium somerae* enhances carbohydrate utilization efficiency in zebrafish through the secretion of acetate [3]. *Clostridium butyricum* can significantly increase the height and width of intestinal villi in largemouth bass while also boosting the activity of key digestive enzymes such as amylase and lipase [4]. *Bacillus subtilis* promotes the growth and development of golden pompano by enhancing the host’s fatty acid β-oxidation capacity and increasing the proportion of beneficial bacteria in the gut [5]. However, many diseases are also closely linked to microorganisms. *Vibrio harveyi* [6] and *Edwardsiella* [7] tarda are typical aquaculture pathogens. When the host is physiologically compromised or environmental conditions deteriorate, commensal bacteria may turn into pathogens. For instance, The abundance of *Aeromonas*, which remained stable initially, increased dramatically after *Macrobrachium rosenbergii* was infected by the parasite *Temnocephala digitata*. Subsequently, *Aeromonas* impaired the host health further by regulating the Toll and Imd signaling pathways [8]. The impact of the gut microbiota on host health depends on its composition and balance: a stable microbial community confers benefits to the host, whereas dysbiosis may pose potential health risks.

Intestinal segments with distinct functions typically harbor specific dominant microbial communities [9]. For instance, in humans, the jejunum—the primary site for nutrient digestion and absorption—is predominantly colonized by *Prevotella* and *Veillonella* [10,11]. The cecum, which contains numerous lymphoid nodules associated with specific immune responses, is mainly inhabited by *Escherichia coli* and *Lactobacillus* [12,13]. The colon, responsible for water absorption and vitamin synthesis, hosts a microbial community dominated by *Bacteroides* and *Faecalibacterium* [14,15,16]. Microbial colonization in the gut depends on the physicochemical properties of the intestinal microenvironment. Studies have confirmed that microorganisms derived from the small intestine tend to colonize the small intestine, while those from the large intestine preferentially colonize the large intestine [17]. Diet is another major factor shaping the gut microbiota; even under similar living conditions, dietary differences can result in distinct microbial compositions. For instance, wild predatory dolphins and captive dolphins fed a fixed diet in the same marine region display clear differences in their gut microbiota: wild individuals are dominated by the phylum Firmicutes, whereas captive individuals are primarily colonized by *Pseudomonadota* (formerly *Proteobacteria*) and *Fusobacteriota* [18].

To improve the economic efficiency of aquaculture and achieve sustainable intensification, the industry is striving to use feeds with higher plant protein content [19] and higher energy density [20], thereby reducing reliance on traditional protein sources such as fishmeal. HFDs are a common type of high-energy feed, offering advantages such as providing essential fatty acids for carnivorous fish growth, promoting the absorption and transport of fat-soluble vitamins, and accelerating growth rates and metabolism. However, excessive feeding of HFDs can also induce adverse effects, such as disruption of the gut microbiota [21], impairment of intestinal barrier function [2], and the development of liver diseases [22].

Nevertheless, current research on intestinal segment-specific microbiota has primarily focused on organisms with well-defined anatomical structures. The characteristics of region-specific microbial distribution in animals lacking distinct intestinal compartmentalization remain understudied. As an important carnivorous species in aquaculture, the largemouth bass possesses an intestinal system characterized by short length, low coiling complexity, and a relatively simple structure. Existing studies lack precise division of its intestinal segments, and the patterns of microbial community changes following HFD intervention remain unclear. We hypothesize that despite the inconspicuous morphological differences and unclear anatomical boundaries between intestinal segments, different regions still tend to exhibit functional specialization, which may lead to microbial enrichment. Based on this hypothesis, we performed rough manual segmentation of the largemouth bass intestine and conducted sequencing, statistical analysis, and evaluation of the microbial communities in different segments. This study presents our novel findings on the dynamics of intestinal microbial communities across segments in largemouth bass under different dietary interventions, revealing microbes with spatial preferences and segment specificity. These findings provide new insights for optimizing the healthy aquaculture of largemouth bass.

## 2. Materials and Methods

### 2.1. Ethical Statement

The entire experimental procedure complied with the Guidelines on the Ethical Treatment of Laboratory Animals (Permit No. DKY-2018202027) issued by the Ministry of Science and Technology of China in 2006. The experimental protocol was approved by the Animal Welfare Advisory Committee of Sichuan Agricultural University.

### 2.2. Diet Formulation

The detailed feed formulations are presented in Table 1. The basal diet contained three protein sources (fish meal, poultry meal, and fermented soybean meal), with soybean oil as the lipid source and two carbohydrate sources (wheat flour and cassava starch) incorporated. An HFD (with a protein level of 49.75% and a lipid level of 17.29%) and a ND (with a protein level of 49.31% and a lipid level of 11.05%) were formulated separately. All feed ingredients were ground, passed through a 40-mesh sieve, and weighed according to the formulated proportions. Micronutrients were added using the stepwise expansion method, thoroughly mixed with the bulk ingredients, and then homogenized again after the addition of oil and water.

### 2.3. Experimental Animal Management and Sample Collection

Juvenile largemouth bass used in the trial were purchased from Qionglai City, Chengdu, Sichuan Province, China. The aquaculture experiment was conducted at the aquaculture base of Ya’an Modern Livestock Science and Technology Innovation Demonstration Park. After a two-week acclimatization period using commercial largemouth bass feed supplied by Tongwei Company (Chengdu, China), 390 healthy largemouth bass with an average initial body weight of 10.87 ± 0.09 g were randomly allocated into 6 square concrete tanks (Length: 2.0 m, Width: 1.5 m, Height: 1.5 m, Water Depth: 1.2 m), with 65 fish per tank. The 6 tanks were randomly divided into 2 treatment groups, with 3 replicates per group. The two treatment groups were fed the ND and HFD, respectively. The growth trial lasted for 10 weeks, during which fish were fed to satiation at 7:00 and 19:00 daily. Residual bait was removed 40 min after each feeding. Random sampling was performed within each replicate at the end of the feeding trial. Weather conditions and water temperature were recorded daily. A continuous flow-through system was adopted, with a water flow rate maintained at 1.0 L/min. After the midday pond inspection, 10 cm of water was exchanged daily. The water temperature during the experimental period was 26.3 ± 1.3 °C. Intermittent aeration was applied to maintain dissolved oxygen levels above 5.0 mg/L.

**Figure 1:**
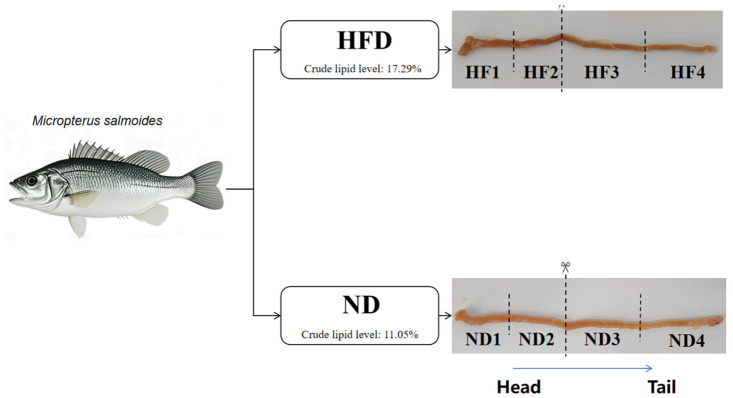
Schematic diagram of dietary groups and intestinal segment sampling in largemouth bass.

Prior to sampling, the experimental fish were fasted for 24 h. Six fish were randomly selected from each replicate and anesthetized with 0.01% MS-222. The largemouth bass were then dissected to collect intestinal samples. From each replicate, intestinal samples from three randomly chosen fish were preserved in 10% neutral buffered formalin for subsequent histological observation. Anatomical examination revealed that the intestine of largemouth bass had one distinct twist, which was used as the primary segmentation point (indicated by scissor marks in Figure 1). The intestine was first cut at this twist, and the resulting two sections were further divided equally by length, yielding a total of four intestinal segments. These four segments were sequentially labeled as segments 1, 2, 3, and 4 from the head to the tail (Figure 1). The segmented intestinal samples were flash-frozen in liquid nitrogen and stored at −80 °C for subsequent bacterial genomic DNA extraction and 16S rRNA gene amplicon sequencing analysis.

### 2.4. Growth Parameters Calculations

The corresponding calculation formula for the growth index is as follows:

Weight gain rate (WGR, %) = (FBW–IBW)/IBW × 100.

Specific growth rate (SGR, %) = Ln (FBW/IBW)/Experimental days × 100.

Survival rate (SR, %) = (finial number of fish)/(initial number of fish) × 100.

Feed conversion rate (FCR, %) = total feed consumption/(FBW–IBW) × 100.

where IBW and FBW were the initial body weight and final body weight.

### 2.5. Intestinal Histological Observation

After fixation in 10% neutral buffered formalin for 24 h, the collected intestinal tissues of largemouth bass were dehydrated through a graded ethanol series, cleared in xylene, immersed in paraffin for 3 h, embedded in paraffin blocks, and sectioned into 4 μm thin slices. The sections were floated in a 42 °C water bath to spread evenly, dried, rehydrated through a graded ethanol series, stained with hematoxylin and eosin (H&E), dehydrated again through a graded ethanol series, and mounted with neutral resin. The prepared sections were scanned using a Zhiyue WS-10 slide scanner, and the scanned images were examined and quantified using Fiji (ImageJ, version 2.9.0/1.54f).

### 2.6. DNA Extraction and PCR Amplification of Intestinal Microbiota

Total genomic DNA was extracted from the intestinal samples using the E.Z.N.A.^®^ Soil DNA Kit (Omega Bio-tek, Norcross, GA, USA). After extraction, DNA integrity was assessed by 1% agarose gel electrophoresis, and DNA concentration and purity were measured using a NanoDrop 2000 spectrophotometer (Thermo Scientific, Waltham, MA, USA). Qualified DNA was used as a template to amplify the V3–V4 hypervariable region of the 16S rRNA gene with the primers 338F (5′-ACTCCTACGGGAGGCAGCAG-3′) and 806R (5′-GGACTACHVGGGTWTCTAAT-3′). PCR products were verified by 1% agarose gel electrophoresis to confirm the amplification of target fragments with the expected size. All amplification products were purified and recovered using the Agencourt AMPure XP nucleic acid purification kit (Beckman Coulter, Brea, CA, USA), and the purified products were quantified using a Qubit 4.0 fluorometer (Thermo Fisher Scientific, Waltham, MA, USA). Library construction was performed with the NEXTFLEX Rapid DNA-Seq Kitusing the purified PCR products, followed by sequencing on the Illumina NextSeq2000 platform.

### 2.7. Sequencing Data Analysis

**Figure 2:**
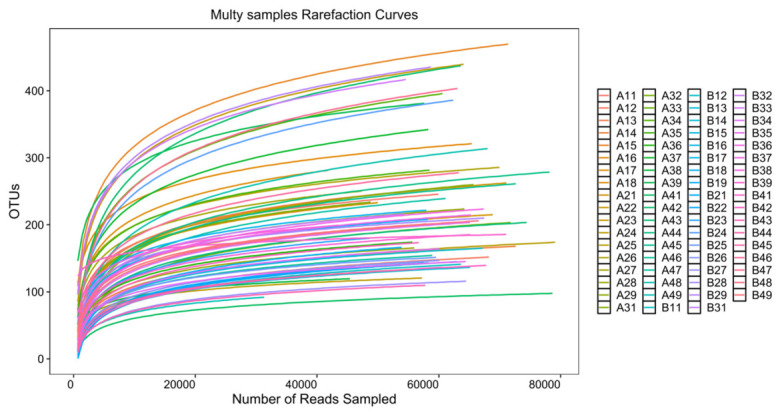
Rarefaction curves of sequencing samples, where A refers to HFD and B refers to ND.

To evaluate the sequencing depth, rarefaction curve analysis was performed, and the results indicated that the current sequencing volume was sufficient to reflect the microbial diversity within the samples (Figure 2).

Raw sequencing data were subjected to quality filtering using Trimmomatic (v0.36) with a sliding window approach, where the window size was set to 50 bp, the average quality threshold to 20, and the minimum retained sequence length to 120 bp. Subsequently, Pear (v0.9.6) was used to remove sequences containing N bases. Based on the processed data, FLASH (v1.2.0) was employed to merge paired-end sequences according to their overlapping regions, with the following parameters: minimum overlap length of 10 bp and maximum mismatch rate of 0.1. This process yielded high-quality sequences after quality control. The sequencing quality statistics are summarized in Supplementary Table S1.

The high-quality sequences were imported into the QIIME 2 platform for subsequent analysis. Using the VSearch plugin, sequences were clustered into operational taxonomic units (OTUs) at a 97% similarity threshold via the UPARSE algorithm. Representative OTU sequences were taxonomically annotated using the feature-classifier plugin in QIIME 2 based on the Silva database. The RDP Classifier algorithm was applied with a confidence threshold of 70% to ensure the reliability of the annotation results. Finally, the annotated results were verified by comparison with the NCBI BLAST database to confirm their accuracy.

### 2.8. Statistical Analysis

All statistical analyses were performed using R software and the QIIME 2 platform. Growth data were compared between the two groups using Student’s *t*-test. Morphological data of intestinal tissues were compared among groups using one-way analysis of variance (ANOVA). Microbial community diversity analysis was conducted on the QIIME 2 platform. Alpha diversity indices were compared between different dietary groups or between different intestinal segments within the same diet using the Wilcoxon rank-sum test. Beta diversity was analyzed based on the Bray-Curtis distance matrix via principal coordinate analysis (PCoA), and the ANOSIM test was used to evaluate the significance of differences in community structure among groups. At the genus level, the Wilcoxon rank-sum test was employed to compare microbial taxa with a relative abundance ≥0.01% in the same intestinal segment between the HFD and ND groups, with *p* values corrected by the Benjamini-Hochberg (BH) approach. To eliminate tank-related confounding effects and avoid pseudoreplication, all phenotypic and histological measurements from individual fish were first averaged at the tank level (three fish per tank), and these tank means were used for subsequent *t*-tests and ANOVA. For microbiome data, alpha diversity indices and genus-level relative abundances were first calculated for each individual sample. These values were then averaged within each tank to produce a single tank-level mean profile. The resulting tank-mean community profiles were used for all subsequent statistical comparisons. For beta diversity, a Bray–Curtis distance matrix was constructed based on these tank-mean profiles, and ANOSIM was performed with tank as the permutation unit. Differences were considered statistically significant when *p* < 0.05.

## 3. Results

### 3.1. Growth Performance

As shown in Table 2, there was no significant difference in initial body weight between the ND and HFD groups. Compared with the ND group, the HFD group showed significantly higher final body weight, weight gain rate (WGR), and specific growth rate (SGR), while the feed conversion ratio (FCR) only exhibited a decreasing tendency without reaching statistical significance. Notably, the survival rate of the HFD group was significantly lower than that of the ND group.

### 3.2. Morphological Characteristics of Intestinal Tissue

**Figure 3:**
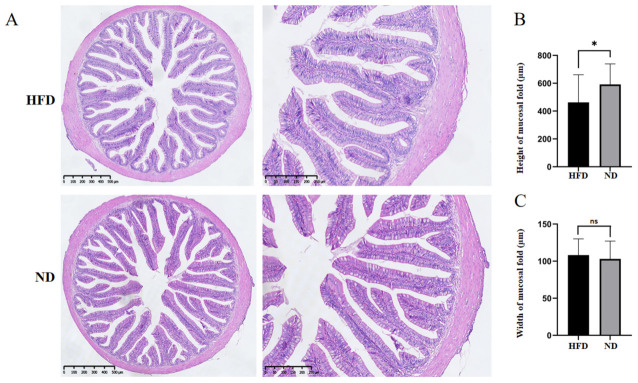
Morphological characteristics of intestinal tissue. H&E staining shows histomorphological differences between the normal diet (ND) and high-fat diet (HFD) groups. (**A**) H&E-stained intestinal sections. scale bars: 500 μm, 250 μm. (**B**) Mucosal fold height and (**C**) width comparisons. Values are mean ± SD (* *p* < 0.05, ns, not significant).

Histological analysis of hematoxylin and eosin (H&E)-stained intestinal tissue paraffin sections revealed the presence of inflammatory necrotic lesions in the intestines of largemouth bass fed the HFD, accompanied by inflammatory cell infiltration around these lesions (Figure 3A). Furthermore, the intestinal fold height of largemouth bass in the HFD group was significantly decreased (Figure 3B; *p* < 0.05), whereas no significant difference in intestinal fold width was observed between the two dietary groups (Figure 3C; *p* > 0.05).

### 3.3. Diversity Changes in Microbial Communities Across Different Intestinal Segments

**Figure 4:**
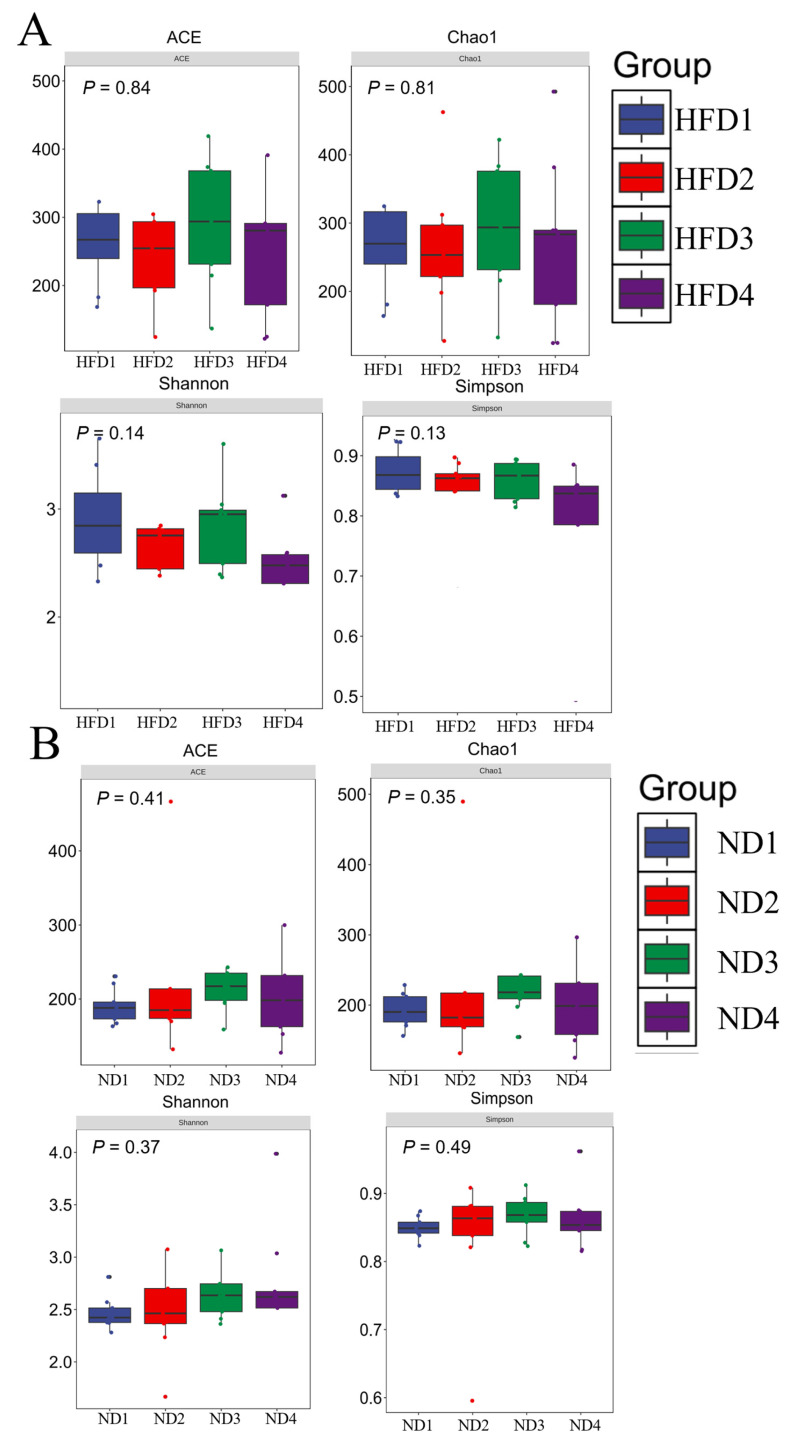
Intestinal segment-specific variations in microbial α-diversity under the same diet. ACE, Chao1, Simpson, and Shannon indices in (**A**) HFD and (**B**) ND groups. Analyzed by Mann–Whitney U test (*p* < 0.05).

We first assessed the differences in alpha diversity indices among the groups and found that none of these indices showed significant differences between different intestinal segments under the same dietary condition (Figure 4; *p* > 0.05).

**Figure 5:**
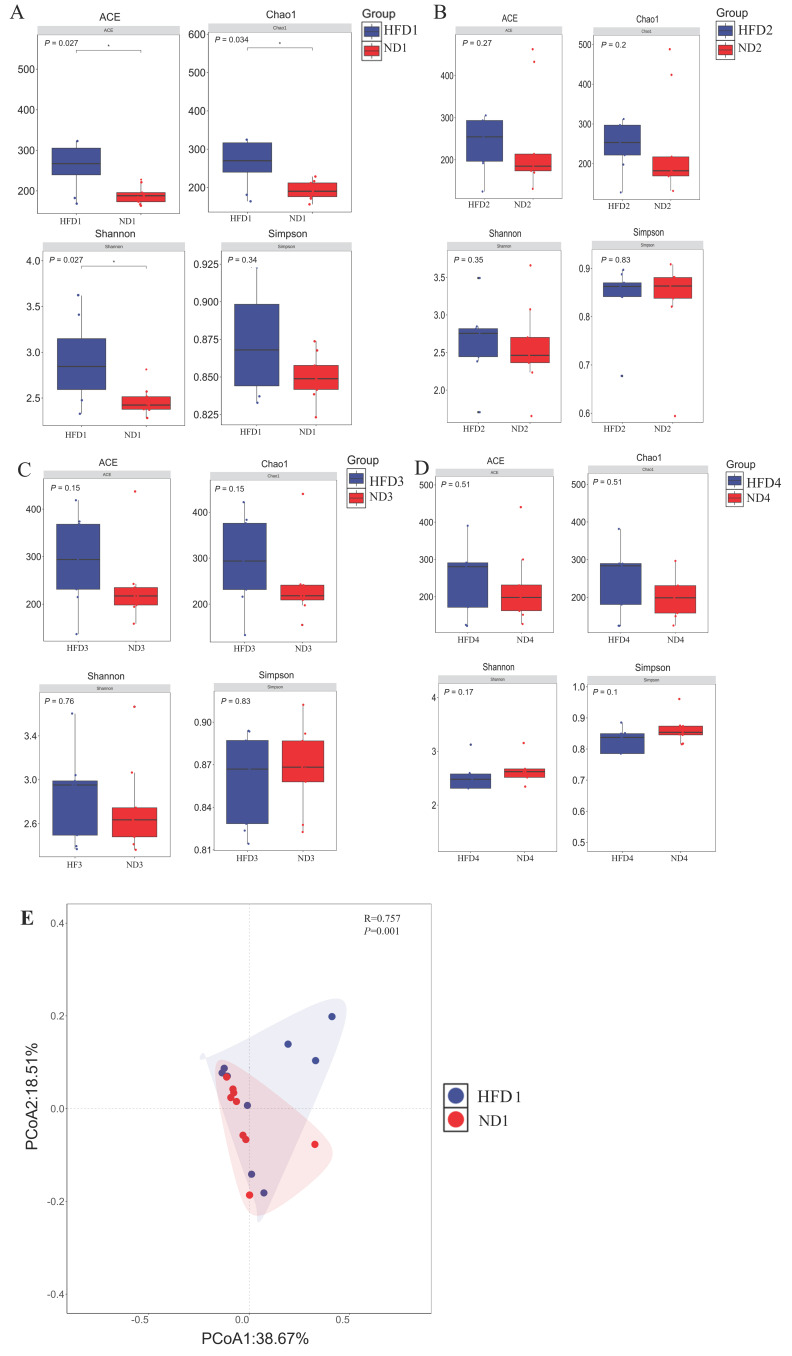
Microbial community diversity changes across intestinal segments under HFD and ND. (**A**–**D**) ACE, Chao1, Simpson, and Shannon indices in segments 1–4, compared between diets (Mann–Whitney U test, *p* < 0.05). (**E**) PCoA plot (Bray–Curtis distance) showing community differences in segment 1. The significance of group differences was determined by ANOSIM (R = 0.757, *p* = 0.001). The calculated data of a single sample are presented in the form of dots in the figure. * *p* < 0.05.

We further compared the differences in alpha diversity indices within the same intestinal segment between the HFD and ND groups. Statistical analysis revealed no significant differences in any of the indices between the HFD and ND groups in intestinal segments 2, 3, and 4 (Figure 5B–D; *p* > 0.05). In contrast, within segment 1, the ACE index, Chao1 index, and Shannon index of the HFD group were significantly higher than those of the ND group (Figure 5A; *p* < 0.05). Principal Coordinate Analysis (PCoA) based on the Bray–Curtis distance metric showed a significant separation between the HFD and ND groups in segment 1 (Figure 5E); PCoA1 explained 38.67% of the total variation, while PCoA2 explained 18.51%. These significant differences were further confirmed by ANOSIM (R = 0.757, *p* = 0.001). Although the differences in the ACE and Chao1 indices between the HFD and ND groups reached statistical significance only in segment 1, these indices were consistently higher in the HFD group than in the ND groups across all examined intestinal segments.

### 3.4. Compositional Changes in Microbial Communities Across Different Intestinal Segments

**Figure 6:**
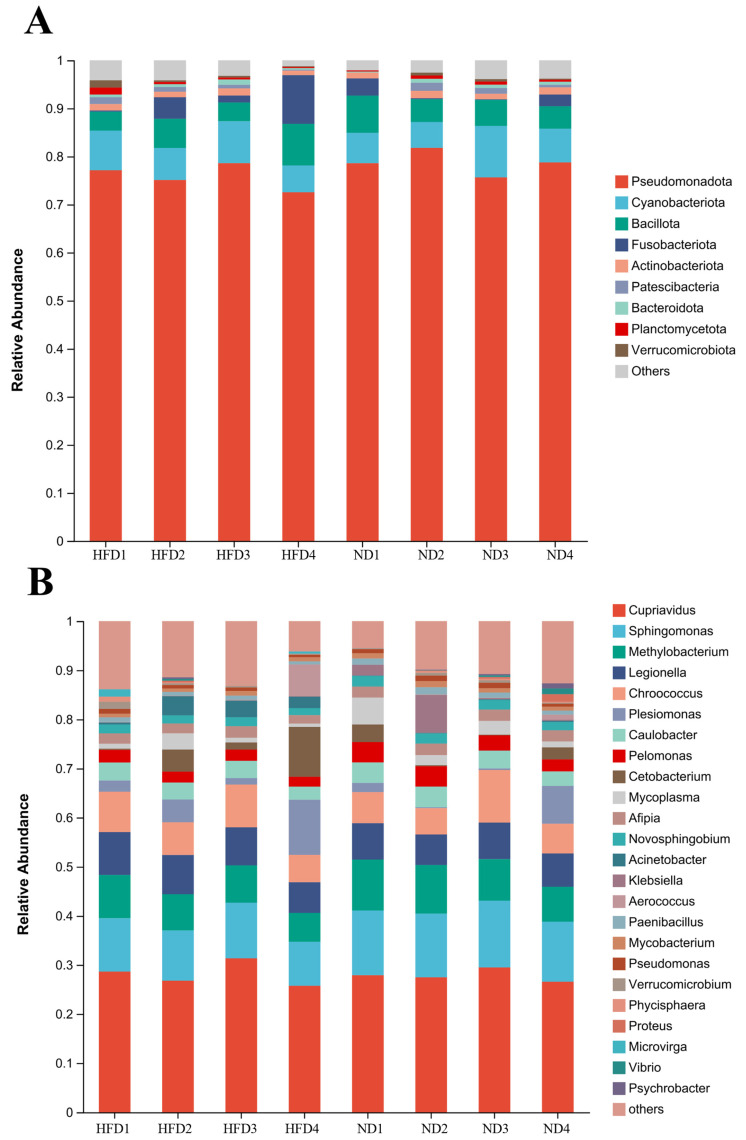
Composition of microbial communities in different intestinal segments. (**A**) Phylum-level distribution (phyla with relative abundance < 1% pooled as Other); (**B**) Genus-level distribution (genera with relative abundance <1% pooled as Other).

Under both dietary conditions, *Pseudomonadota* was the dominant microbial phylum across all intestinal segments, accounting for more than 70% of the total relative abundance. Other phyla with relatively high abundances, in descending order, were *Cyanobacteria*, *Bacillota* (formerly *Firmicutes*), *Fusobacteriota*, and *Actinobacteriota* (Figure 6A). At the genus level, the most abundant microorganism was *Cupriavidus*, followed by *Sphingomonas*, *Methylobacterium*, and *Legionella* (Figure 6B). Among the genera with a relative abundance above 1%, some exhibited distinct spatial distribution preferences, being concentrated in specific intestinal segments. For instance, *Plesiomonas*, *Aerococcus*, and *Cetobacterium* were predominantly enriched in segment 4, with relative abundances of 11.25%, 6.47%, and 10.14% in the HFD group, and 7.68%, 1.02%, and 2.44% in the ND group, respectively—all of which were markedly higher than those in the other three segments. *Klebsiella* was primarily enriched in segments 1 and 2 of the ND group, while its relative abundance was low across all segments in the HFD group. The relative abundance of *Acinetobacter* was lower only in segment 1, and this reduction was more pronounced under the HFD condition. The relative abundance of *Pelomonas* in each segment of the HFD group was lower than that in the ND group.

**Figure 7:**
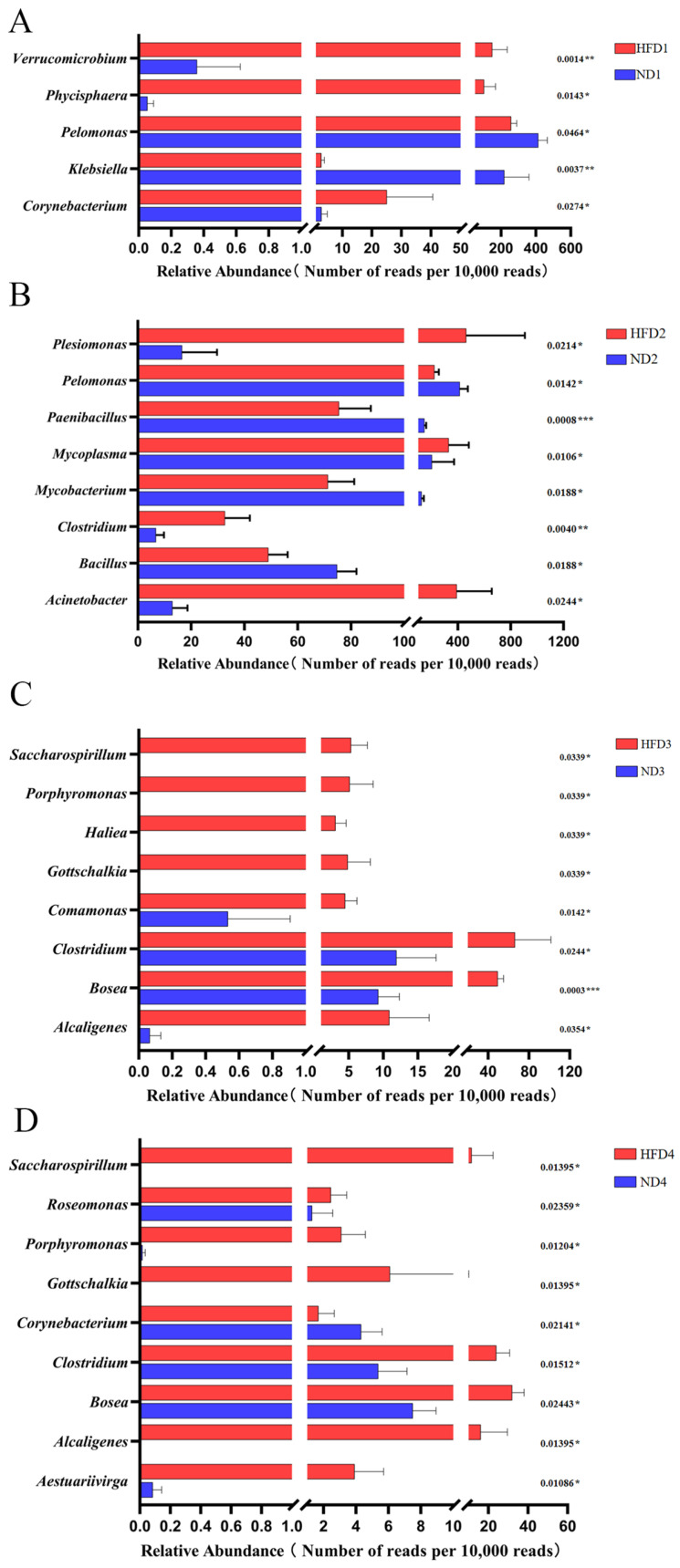
Wilcoxon rank-sum test of microorganisms in different intestinal segments. Comparisons of microbial genera between HFD and ND in largemouth bass intestinal segments. Only genera with relative abundance > 0.01% were analyzed. (**A**–**D**) show genera with significantly differential abundances in the intestinal segment 1, 2, 3 and 4. Values are mean ± SEM. * *p* < 0.05, ** *p* < 0.01, *** *p* < 0.001.

To further understand the changes in microbial composition across different intestinal segments, we performed Wilcoxon rank-sum tests on the relative abundances of all bacterial genera detected in each segment to analyze their differences between the HFD and ND groups. Statistically, intestinal microorganisms with a relative abundance ≥ 0.01% were clustered into 245 operational taxonomic units (OTUs), which belonged to 141 distinct genera. The results of the Wilcoxon rank-sum test showed that the microbial taxa with significant differences were not identical across intestinal segments (*p* < 0.05). Under HFD intervention, five genera exhibited significant alterations in the first intestinal segment (*p* < 0.05): the relative abundances of *Pelomonas* and *Klebsiella* decreased significantly, while those of the other three genera increased significantly (Figure 7A). In the second intestinal segment, eight genera showed significant changes (*p* < 0.05): the relative abundances of *Pelomonas*, *Paenibacillus*, *Mycobacterium*, and *Bacillus* decreased significantly, while those of the remaining four genera increased significantly (Figure 7B). In the third intestinal segment, eight genera were significantly different (*p* < 0.05), all of which showed increased relative abundances (Figure 7C). In the fourth intestinal segment, nine genera exhibited significant differences (*p* < 0.05): the relative abundance of *Corynebacterium* decreased significantly, while those of the other eight genera increased significantly (Figure 7D).

### 3.5. Diversity Changes in the Microbial Community of the Whole Intestine

**Figure 8:**
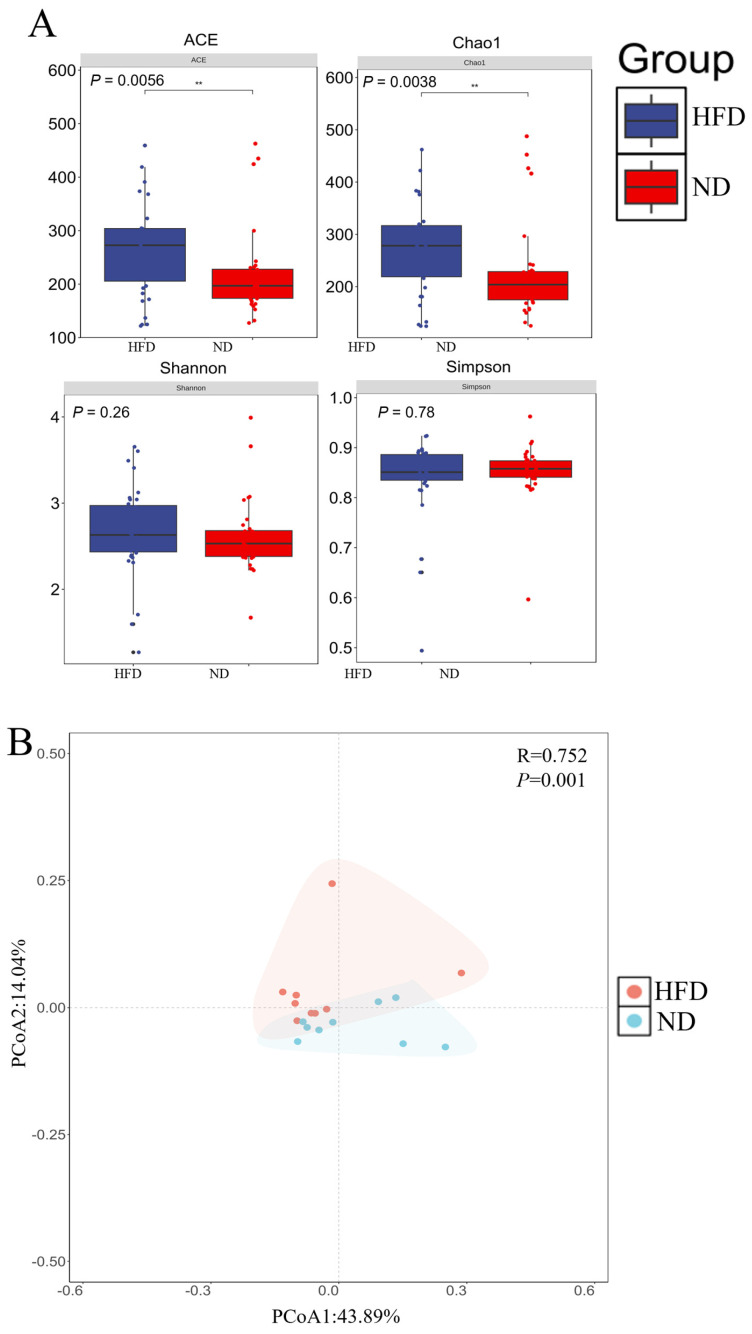
Changes in microbial community diversity of the whole intestine. (**A**) ACE, Chao1, Shannon, and Simpson of intestinal microbes under HFD and ND (Mann–Whitney U test, ** *p* < 0.01). (**B**) PCoA plot based on Bray–Curtis distance; significance determined by ANOSIM (R = 0.752, *p* = 0.001). The calculated data of a single sample are presented in the form of dots in the figure.

Previous studies on the intestinal microbiota of largemouth bass have typically treated the intestine as a whole. To clarify the differences between these analytical approaches, this study also adopted a whole-intestine analysis strategy to evaluate the microbial composition within the same set of samples. Statistical analysis of all intestinal data from the HFD and ND groups showed that the ACE and Chao1 indices in the HFD group were significantly higher than those in the ND group (Figure 8A). PCoA based on the Bray–Curtis metric revealed a clear separation between the intestinal microbial communities of the two groups (Figure 8B), with PCo1 and PCo2 explaining 43.89% and 14.04% of the total variation, respectively. ANOSIM further confirmed these significant differences (R = 0.752, *p* = 0.001).

### 3.6. Compositional Changes in the Microbial Community of the Whole Intestine

**Figure 9:**
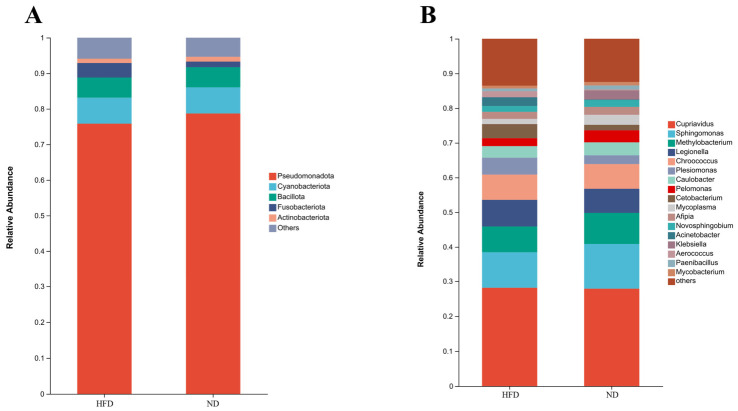
Changes in microbial community composition of the whole intestine. (**A**) Phylum-level distribution (phyla with relative abundance <1% pooled as Other); (**B**) Genus-level distribution (genera with relative abundance <1% pooled as Other).

At the phylum level, *Pseudomonadota*, *Cyanobacteriota*, *Bacillota*, *Fusobacteriota*, and *Actinobacteriota* were the phyla with relative abundances exceeding 1% in the whole intestine. Among them, *Pseudomonadota* was absolutely dominant, accounting for 75.83% and 78.69% in the HFD group and ND group, respectively. Under high-fat diet stimulation, only *Fusobacteriota* showed a substantial increase in relative abundance, rising from 1.58% under the ND to 4.07% under the high-fat diet (Figure 9A). In addition, genera such as *Plesiomonas*, *Klebsiella*, *Acinetobacter*, and *Aerococcus*, which exhibited noticeable changes in relative abundance in the segment-based analysis, showed only minor variations of 1.18%, 2.54%, 2.50%, and 2.36% (Figure 9B), respectively, from the whole-intestine perspective. Such subtle differences can easily be overlooked in an overall analysis.

**Figure 10:**
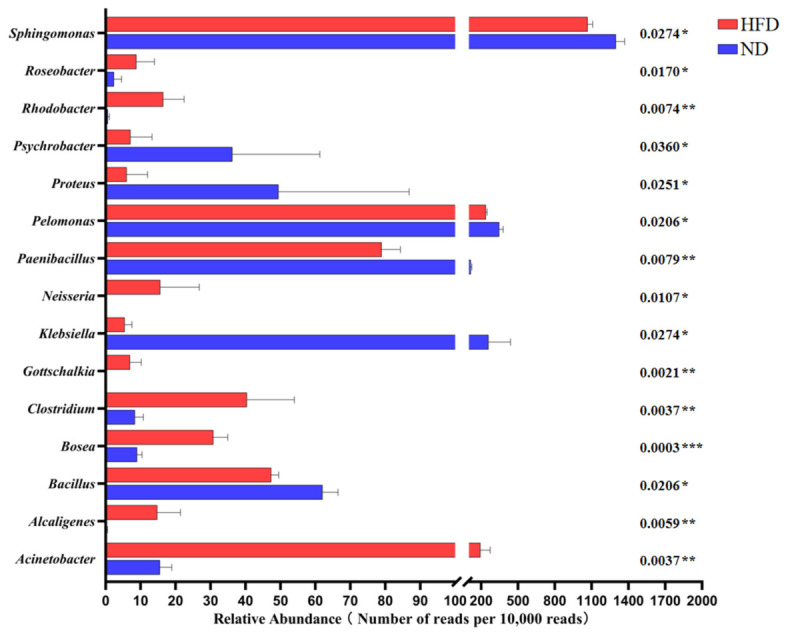
Wilcoxon rank-sum test of whole intestinal microbiota. Microbial genera with significant differences between HFD and ND groups; genera with relative abundance < 0.01% were excluded. Values are mean ± SEM. * *p* < 0.05, ** *p* < 0.01, *** *p* < 0.001.

Wilcoxon rank-sum tests on microbial genera with relative abundances above 0.01% in the whole intestine under high-fat diet and ND conditions revealed 15 genera with significant differences (*p* < 0.05; Figure 10). Compared with the results presented in Section 3.3, 12 genera—*Aestuariivirga*, *Corynebacterium*, *Comamonas*, *Haliea*, *Mycobacterium*, *Mycoplasma*, *Plesiomonas*, *Phycisphaera*, *Porphyromonas*, *Roseomonas*, *Saccharospirillum*, and *Verrucomicrobium*—were detected as significantly different only in the segment-based analysis. In contrast, only five genera—*Neisseria*, *Psychrobacter*, *Proteus*, *Rhodobacter*, and *Roseobacter*—showed significant differences exclusively in the whole-intestine analysis. Overall, although treating the intestine of largemouth bass as a homogeneous unit can provide an overview of the intestinal microbial community, this approach tends to overlook the inherent spatial heterogeneity of the gut, making it difficult to analyze regional variations in the microbiota and potentially missing microbial changes that occur only in specific intestinal segments.

## 4. Discussion

HFDs have attracted considerable attention in aquaculture due to their advantages of reducing feed costs, supplying essential fatty acids, and promoting fish growth [23]. However, excessive fat intake is associated with adverse effects, including impaired lipid metabolism and immunosuppression [24]. This is consistent with the results of the present feeding trial, where the HFD group exhibited better growth performance but higher mortality, indicating compromised health status in largemouth bass fed HFD. Currently, it is widely recognized that an HFD can alter the structure of the gut microbiota, which has been supported by studies in both mammals [25,26] and fish [21,27]. Nevertheless, the regulatory pattern of gut microbiota in response to an HFD remains poorly understood in largemouth bass. In this study, we integrated histological observation of intestinal morphology and 16S rRNA gene sequencing to analyze the dynamic changes in microbial communities across different intestinal segments of largemouth bass fed ND and HFD.

### 4.1. Alterations in Intestinal Histomorphology

Intestinal mucosal folds are formed by the protrusion of both the mucosal and submucosal layers into the intestinal lumen. These folds increase the intestinal contact surface area, thereby facilitating nutrient absorption [28]. Under HFD intervention, the height of intestinal folds in largemouth bass decreased significantly, while their width showed no significant change. This reduction in fold surface area likely impairs the intestine’s capacity for nutrient absorption. Previous studies have indicated that HFDs may coincide with intestinal inflammation [1,24]. Consistent with this finding, our observations revealed the presence of inflammatory necrotic foci accompanied by inflammatory cell infiltration in the intestines of fish fed the HFD. These results suggest that intestinal homeostasis in largemouth bass was disrupted, prompting us to investigate the associated changes in the gut microbiota.

We speculate that there are two potential bidirectional associations between HFD-induced intestinal tissue damage and gut microbiota dysbiosis in fish. On one hand, an HFD directly may coincide with intestinal lipotoxicity, potentially linked to intestinal tissue lesions and microbiota imbalance, which in turn may be associated with elevated disease susceptibility and mortality in fish. On the other hand, an HFD first disturbs host metabolism and impairs overall physiological functions and disease resistance, with intestinal damage and gut microbiota dysbiosis occurring as secondary manifestations. These two mechanisms are not mutually exclusive; instead, they may form a mutually correlated feedback loop. Specifically, the HFD initially damages intestinal morphology and disrupts microbial composition via primary effects. The impaired intestinal barrier and disrupted microecology further suppressed immune and digestive functions, concurrent systemic health disorders and higher mortality risk. In turn, the declined physiological status and poor health of fish may be potentially linked to more severe intestinal inflammation and microbiota dysbiosis. The cumulative effects of these multiple factors ultimately may be associated with severe intestinal lesions, substantial gut microbiota perturbation and a marked reduction in survival rate in fish fed the HFD.

### 4.2. Advantages of Segmentation in Research

Segmenting intestinal tracts for research purposes is a well-established practice, and studies involving gut microbiota typically specify the exact intestinal segment [17,29,30]. In the absence of clear anatomical partitioning in the intestinal tract of largemouth bass, this study relied on observable structures and length to perform artificial division. We then compared the differences in microbial diversity and composition across these intestinal segments between the HFD and ND groups. The results showed that, from a segmented perspective, the relative abundance of 21 microbial genera changed significantly (*p* < 0.05). In contrast, from an overall intestinal perspective, only 15 genera showed significant differences in relative abundance (*p* < 0.05). Crucially, only the segmented analysis revealed the significant proliferation of typical opportunistic potentially pathogenic taxa such as *Mycoplasma*, *Mycobacterium*, and *Roseomonas* induced by the HFD. Furthermore, genera like *Plesiomonas* and *Klebsiella*, which exhibited high abundance in specific segments, could be easily overlooked in statistical analysis under the overall perspective due to their less prominent overall relative abundance. These findings indicate that, compared to a holistic approach, a segmented study can unveil richer microbial information and provide a more comprehensive analytical perspective. Simultaneously, investigating the association between the gut microbiota and intestinal functions can offer a more complete theoretical basis for scientifically delineating the intestinal system of largemouth bass.

However, the method of dividing and grouping intestinal segments based on visual observation and length also has certain limitations. This approach is relatively coarse and represents an attempt made in the context of the absence of clear anatomical demarcation in the intestinal tract of largemouth bass. Future studies that can more clearly define the structural partitions of its intestine will help overcome the shortcomings of the current method.

### 4.3. Microbial Community Composition Varies Across Intestinal Segments

By conducting refined segmentation of the intestinal system, we found that various gut microbes exhibited distinct spatial distribution preferences, with significant differences in microbial communities among different intestinal segments. These results strongly support our hypothesis that although the intestinal structure of largemouth bass is not distinctly differentiated, the spatial preference exhibited by its microbial community suggests potential functional enrichment in different intestinal segments. The study demonstrated that several microbial genera tended to be enriched in specific intestinal segments under both HFD and ND conditions. For example, *Klebsiella* was primarily enriched in segments 1 and 2, while *Plesiomonas*, *Aerococcus*, and *Cetobacterium* were mainly enriched in segment 4; in contrast, *Acinetobacter* maintained a relatively high abundance in all segments except segment 1, where its level was lower. These microbial genera have distinct functions: Although *Klebsiella* is classified as an opportunistic pathogen, under conditions of normal microecological homeostasis, it possesses the ability to metabolize diverse carbohydrates, thereby assisting the host in carbohydrate digestion and providing fermentation substrates for other microorganisms [31,32]; *Cetobacterium* can secrete acetate and synthesize vitamin B12 [3], which facilitates the host’s transformation of carbohydrates, fats, and proteins, suggesting that the function of segment 4 may be related to metabolic regulation after nutrient absorption; *Acinetobacter* has lipid metabolism capabilities [33] and is widely distributed in all intestinal segments except segment 1. This indicates that most intestinal regions of the carnivorous largemouth bass may have the potential for lipid absorption, and the abundant lipid nutrients provided by the HFD may have promoted its proliferation.

Based on inferences from intestinal physiological functions, Intestinal Segment 1 serves as the initial site for food entry into the intestine, where carbohydrates remain insufficiently digested. The carbohydrate metabolic capacity of *Klebsiella* facilitates its enrichment in this segment, implying that Intestinal Segment 1 may suggest that this region is potentially associated with carbohydrate digestion in largemouth bass. As the distal part of the intestine, As the distal part of the intestine, Intestinal Segment 4 is potentially associated with major nutrient absorption; *Cetobacterium* secretes acetic acid to participate in the host’s metabolic regulation. Furthermore, *Acinetobacter* has lipid metabolic capacity and exhibits high abundance in all intestinal segments except Intestinal Segment 1, which is consistent with the lipid absorption requirements of carnivorous largemouth bass. The distinct functions of each intestinal segment are potentially associated with microbial spatial distribution and require further validation.

Segment 1 was the only segment where the changes in the ACE index and Chao1 index reached statistical significance under HFD intervention, indicating that the microbial richness in this segment increased the most and was most affected by the HFD. This is likely because segment 1, as the initial site of food entry, may experience the highest lipid load, bile acid concentration, or oxidative stress, which could explain the most significant changes in microbial richness.

### 4.4. High-Fat Diet Potentially Associated with Alterations in Gut Microbiota

The HFD was accompanied by alterations in the gut microbiota of largemouth bass. In this study, under high-fat diet intervention, both the ACE and Chao1 indices of the microbiota tended to increase across all intestinal segments, indicating an overall enhancement of microbial richness. At the phylum level, the change in *Fusobacteriota* was the most apparent, particularly in the fourth intestinal segment. The over-proliferation of *Fusobacteriota* may be potentially linked to markers of compromised intestinal health in largemouth bass. Furthermore, the significant proliferation of multiple microbial genera following HFD intervention may be correlate with the host’s reduced efficiency in utilizing energy from the HFD, leading to decreased digestive efficiency. Undigested nutrients then provide a richer substrate for microorganisms, promoting their growth.

The relative abundances of multiple pathogenic and probiotic bacteria also changed notably under HFD intervention. At the genus level, we found significant changes (*p* < 0.05) in the relative abundances of various opportunistic potentially pathogenic taxa in the intestine. For instance, *Acinetobacter* and *Plesiomonas* [34] are common opportunistic potentially pathogenic taxa whose abnormal proliferation is often associated with immunosuppression and inflammation, and was significantly correlated with the inflammatory necrotic foci observed in this study. Other conditionally pathogenic bacteria with significantly increased relative abundance included *Staphylococcus*, *Peptostreptococcus*, and *Mycoplasma*. Dysbiosis manifests in diverse forms, and not all opportunistic pathogens exhibited proliferation [35]. For example, although *Klebsiella* is an opportunistic pathogen, it possesses the ability to metabolize various saccharides, facilitating host carbohydrate digestion and providing fermentation substrates for other microbes [36]. Under HFD conditions, the relative abundance of *Klebsiella* decreased significantly, and this sharp change itself indicates disruption of the original microecological homeostasis. Additionally, the HFD also was associated with a significant reduction in certain probiotics. Genera such as *Paenibacillus* and *Bacillus*, which can enhance intestinal barrier function [37,38] and secrete antimicrobial substances [39,40], showed markedly decreased relative abundances under HFD conditions. The weakened antagonistic effect against opportunistic potentially pathogenic taxa may be potentially associated with the substantial proliferation of multiple such potentially pathogenic taxa under HFD intervention.

Accordingly, we speculate that an HFD can directly may coincide with morphological damage and localized inflammatory responses in largemouth bass, accompanied by alterations in the intestinal microbial community structure. Long-term excessive intake of an HFD further may correlate with worsened the physiological health status of fish, which is potentially linked to enhanced the pathogenic potential of opportunistic potentially pathogenic taxa [34,41,42]. These potentially pathogenic taxa are present at low abundance or remain non-pathogenic under healthy physiological conditions. Collectively, these two adverse factors may form a vicious cycle, synergistically potentially linked to more severe intestinal microecological dysbiosis and intestinal inflammation. Ultimately, this cascade of events may be associated with comprehensive impairment of intestinal function and a marked increase in the mortality rate of largemouth bass.

When designing the animal trial, bentonite was incorporated as a filler to balance dietary lipid contents, and varying inclusion levels of bentonite may interfere with microbial sequencing results. It is undeniable that 16S rRNA gene sequencing has inherent limitations. Taxonomic annotation is only reliable at the genus level, which fails to differentiate pathogenic and non-pathogenic strains within the same genus. Accordingly, the correlation analyses of microbial abundance shifts, pathogen identification and host health status in this study were comprehensively deduced based on existing aquaculture research. In future work, combined multi-omics approaches will be adopted to achieve more accurate investigations.

## 5. Conclusions

In summary, this study demonstrates spatial distribution preferences of certain microbial genera in the intestinal tract of largemouth bass. *Klebsiella*, which is capable of metabolizing carbohydrates, was primarily enriched in the first and second intestinal segments, while *Cetobacterium*, known for producing acetic acid, was predominantly enriched in the fourth intestinal segment. In contrast, *Acinetobacter*, which metabolizes lipids, showed a relatively high abundance across most intestinal segments. The functions of these microbes may be coupled with the physiological roles of their respective intestinal segments. Moreover, an HFD significantly increased the microbial richness in the first intestinal segment and induced adverse alterations in intestinal tissue morphology. The relative abundances of several microbial genera were markedly altered by the HFD, with opportunistic potentially pathogenic taxa such as *Acinetobacter* and *Plesiomonas* showing significant proliferation, while beneficial bacteria including *Paenibacillus* and *Bacillus* were notably reduced.

This study provides insights into the response characteristics of the intestinal microbiota in largemouth bass to high-fat intervention from a segment-specific perspective, offering a theoretical basis for future targeted nutritional regulation and optimization of healthy aquaculture strategies.

## Figures and Tables

**Table 1:** Formulation and proximate composition (%) of the experimental diets.

Ingredients (% Dry Matter)	HFD	ND
soybean oil	12.00	6.00
Bentonite ^a^	1.00	7.00
Fish Meal ^b^	43.00	43.00
Chicken Meal ^c^	16.00	16.00
Vital Wheat Gluten ^d^	7.00	7.00
Fermented soybean meal	5.00	5.00
Wheat Flour ^e^	6.00	6.00
Cassava Starch ^f^	5.70	5.70
Lysine	0.20	0.20
Methionine	0.20	0.20
Monocalcium ^g^	1.00	1.00
Choline chloride	0.40	0.40
Minerals ^h^	2.50	2.50
Nutritional components (%)		
Crude protein	49.75	49.31
Crude fat	17.29	11.05
Moisture content	6.88	6.64
Ash	5.52	9.61

^a^ Bentonite: Guanghan Longteng Mining Co., Ltd. (Guanghan, China); ^b^ Fish Meal: TASA (Lima, Peru); ^c^ Chicken Meal: Dalian Xinyuan Feed Co., Ltd. (Dalian, China); ^d^ Vital Wheat Gluten: Dongguan Yihai Kerry Sairui Starch Co., Ltd. (Dongguan, China); ^e^ Wheat Flour: Sichuan Zaishengyuan Flour Co., Ltd. (Deyang, China); ^f^ Cassava Starch: Honghe Shanluo Agricultural Products Processing Co., Ltd. (China); ^g^ Monocalcium Phosphate: Kunming Chuanjinnuo Chemical Co., Ltd. (China); ^h^ Minerals (mg/kg) and Vitamins (IU or mg/kg) Premix: FeSO_4_·H_2_O, 100.00 mg; MgSO_4_·H_2_O, 625.00 mg; CuSO_4_·5H_2_O, 20 mg; ZnSO_4_·H_2_O, 115.94 mg; MnSO_4_·H_2_O, 37.74 mg; CoCl_2_·6H_2_O, 81.97 mg; Ca(IO_3_)_2_, 61.35 mg; Na_2_SeO_3_, 200 mg; KCl, 95.60 mg; NaCl, 76.26 mg; Vitamin A, 133.33 mg; Vitamin E, 220.00 mg; Vitamin B12, 100.00 mg; Vitamin D3, 2.40; Folic acid, 2.00 mg; Biotin, 50.00 mg; Inositol, 408.16 mg; Vitamin B1, 21.00 mg; Vitamin B2, 43.75 mg; Vitamin B6, 22.00 mg; Vitamin K3, 23.23 mg; Pantothenic acid, 37.76 mg; Vitamin C, 157.89 mg.

**Table 2:** High-fat diets promote growth performance in largemouth bass.

Item	ND	HF	*p*-Value
IBW (g/fish)	10.87 ± 0.04	10.87 ± 0.05	0.9985
FBW (g/fish)	73.21 ± 3.97 ^a^	82.78 ± 7.64 ^b^	0.0307
WGR (%)	573.82 ± 38.81 ^a^	661.56 ± 60.34 ^b^	0.0341
SGR (%/d)	2.61 ± 0.08 ^a^	2.77 ± 0.19 ^b^	0.0415
FCR (%)	1.51 ± 0.11 ^a^	1.31 ± 0.12 ^b^	0.0668
SR (%)	100 ± 0.00 ^a^	86.22 ± 1.55 ^b^	0.0018

Note: IBW = Initial mean body weight, FBW = Final body weight, WGR = Weight gain rate, SGR = Specific growth rate, FCR = Feed conversion ratio, SR = Survival rate. Data represent the means ± SD of each group. *p* < 0.05 represents significant difference. Means in the same row marked with distinct superscript letters (a, b) differ significantly at *p* < 0.05.

## Data Availability

The datasets generated and analyzed during this study are available from the corresponding author upon reasonable request.

## Supplementary Materials

The following supporting information can be downloaded at: https://www.mdpi.com/article/10.3390/ani16142141/s1. The supplementary data of this paper include one table, which is used to supplement and explain detailed sequencing information of the experiment. Supplementary Table S1 displays the quality statistics of 16S rRNA amplicon sequencing data of largemouth bass intestine, reflecting reliable sequencing data quality for downstream analysis..
