# Supplementary material for: Effects of High-Fat Diet on Intestinal Microbiota in Largemouth Bass (Micropterus salmoides) from a Segmental Perspective
Source: Animals (Basel). 2026 Jul 9;16(14):2141. doi: 10.3390/ani16142141 (PMC13405785; doi:10.3390/ani16142141)
Supplement: Supplementary file 1 [file animals-16-02141-s001.zip › animals-4342586-supplementary.pdf]

Supplementary Material

Table S1. Quality statistics of 16S rRNA amplicon sequencing data of largemouth bass intestine

sample	group	Raw reads	Clean reads	Effective	Q20%	Q30%	GC%	goods_coverage
HF1_1a	HFD2	72939	68108	93.38%	98.47%	94.72%	54.52%	99.88%
HF1_1b	HFD4	83079	76474	92.05%	98.42%	94.41%	50.97%	99.95%
HF1_2a	HFD2	74939	70333	93.85%	98.48%	94.69%	54.36%	99.84%
HF1_2b	HFD4	69124	64658	93.54%	98.43%	94.57%	54.29%	99.70%
HF1_3a	HFD2	70824	66456	93.83%	98.47%	94.77%	54.31%	99.76%
HF1_3b	HFD4	79183	74525	94.12%	98.41%	94.48%	54.38%	99.88%
HF1_4a	HFD1	66573	58803	88.33%	97.79%	92.70%	54.51%	99.85%
HF1_4b	HFD3	70497	66378	94.16%	98.48%	94.66%	54.71%	99.81%
HF1_5a	HFD1	70967	66382	93.54%	98.49%	94.73%	54.71%	99.88%
HF1_5b	HFD3	53656	49868	92.94%	98.33%	94.31%	53.44%	99.88%
HF1_6a	HFD1	71366	67194	94.15%	98.52%	94.79%	54.48%	99.85%
HF1_6b	HFD3	74807	69950	93.51%	98.36%	94.41%	54.64%	99.87%
HF2_1a	HFD2	60758	56881	93.62%	98.41%	94.49%	52.09%	99.95%
HF2_1b	HFD4	43580	40774	93.56%	98.46%	94.64%	54.54%	99.82%
HF2_2a	HFD2	82262	77208	93.86%	98.48%	94.72%	54.65%	99.88%
HF2_2b	HFD4	76920	72319	94.02%	98.47%	94.67%	54.14%	99.79%
HF2_3a	HFD2	74798	70021	93.61%	98.48%	94.78%	54.66%	99.88%
HF2_3b	HFD4	61658	57950	93.99%	98.45%	94.66%	54.84%	99.92%
HF2_4a	HFD1	75370	70764	93.89%	98.44%	94.62%	54.54%	99.90%
HF2_4b	HFD3	67430	63138	93.63%	98.40%	94.55%	54.32%	99.78%
HF2_5a	HFD1	80422	75606	94.01%	98.49%	94.79%	54.41%	99.79%
HF2_5b	HFD3	64307	60819	94.58%	98.48%	94.68%	53.62%	99.87%
HF2_6a	HFD1	73690	68855	93.44%	98.49%	94.73%	54.26%	99.89%
HF2_6b	HFD3	58512	55068	94.11%	98.48%	94.69%	54.36%	99.89%
HF3_1a	HFD2	52850	49325	93.33%	98.44%	94.63%	54.57%	99.88%
HF3_1b	HFD4	64364	60431	93.89%	98.40%	94.53%	54.90%	99.82%
HF3_2a	HFD2	58762	55160	93.87%	98.43%	94.60%	53.51%	99.88%
HF3_2b	HFD4	73445	69307	94.37%	98.40%	94.57%	53.96%	99.74%
HF3_3a	HFD2	68717	64694	94.15%	98.46%	94.64%	53.28%	99.86%
HF3_3b	HFD4	48119	46092	95.79%	98.29%	94.24%	54.39%	99.92%
HF3_4a	HFD1	47333	44315	93.62%	98.39%	94.45%	54.37%	99.87%
HF3_4b	HFD3	65304	61157	93.65%	98.43%	94.61%	54.69%	99.81%
HF3_5a	HFD1	55446	51650	93.15%	98.43%	94.61%	54.59%	99.89%
HF3_5b	HFD3	48036	44837	93.34%	98.47%	94.69%	54.24%	99.96%
HF3_6b	HFD3	66214	61984	93.61%	98.38%	94.50%	54.51%	99.92%
NC1_1a	ND2	65075	61124	93.93%	98.49%	94.76%	54.14%	99.88%
NC1_1b	ND4	42282	39266	92.87%	98.33%	94.41%	53.09%	99.97%
NC1_2a	ND2	60539	57434	94.87%	98.36%	94.53%	55.49%	99.87%

NC1_2b	ND4	72583	68154	93.90%	98.52%	94.82%	53.78%	99.87%
NC1_3a	ND2	51196	48153	94.06%	98.53%	94.88%	54.46%	99.91%
NC1_3b	ND4	59832	56132	93.82%	98.50%	94.79%	54.66%	99.90%
NC1_4a	ND1	66088	62130	94.01%	98.42%	94.56%	54.87%	99.89%
NC1_4b	ND3	59552	55542	93.27%	98.50%	94.81%	54.41%	99.88%
NC1_5a	ND1	70645	66097	93.56%	98.52%	94.83%	53.13%	99.89%
NC1_5b	ND3	61019	57318	93.93%	98.44%	94.68%	54.33%	99.82%
NC1_6a	ND1	67509	63267	93.72%	98.38%	94.43%	55.04%	99.87%
NC1_6b	ND3	68533	64453	94.05%	98.53%	94.85%	54.67%	99.88%
NC2_1a	ND2	66854	62574	93.60%	98.47%	94.75%	54.38%	99.76%
NC2_1b	ND4	59787	56078	93.80%	98.47%	94.77%	54.82%	99.90%
NC2_2a	ND2	62298	58646	94.14%	98.46%	94.68%	54.75%	99.91%
NC2_2b	ND4	57966	54633	94.25%	98.43%	94.66%	54.56%	99.92%
NC2_3a	ND2	62292	58696	94.23%	98.54%	94.85%	54.84%	99.89%
NC2_3b	ND4	61944	57516	92.85%	98.32%	94.34%	54.08%	99.83%
NC2_4a	ND1	60235	56326	93.51%	98.34%	94.36%	52.93%	99.89%
NC2_4b	ND3	62349	58530	93.87%	98.50%	94.79%	54.60%	99.89%
NC2_5a	ND1	48236	45197	93.70%	98.43%	94.63%	54.70%	99.86%
NC2_5b	ND3	67434	62833	93.18%	98.36%	94.43%	53.79%	99.92%
NC2_6a	ND1	60542	56955	94.08%	98.50%	94.78%	54.56%	99.90%
NC2_6b	ND3	68920	64538	93.64%	98.52%	94.81%	54.42%	99.90%
NC3_1a	ND2	66691	62689	94.00%	98.52%	94.82%	54.88%	99.91%
NC3_1b	ND4	67540	63307	93.73%	98.33%	94.32%	54.55%	99.73%
NC3_2a	ND2	72140	67546	93.63%	98.46%	94.71%	55.11%	99.91%
NC3_2b	ND4	72522	68325	94.21%	98.42%	94.61%	54.62%	99.89%
NC3_3a	ND2	67486	63445	94.01%	98.46%	94.73%	54.03%	99.78%
NC3_3b	ND4	61211	57503	93.94%	98.49%	94.70%	54.33%	99.89%
NC3_4a	ND1	62146	58441	94.04%	98.54%	94.89%	54.35%	99.89%
NC3_4b	ND3	71019	66490	93.62%	98.30%	94.14%	54.76%	99.84%
NC3_5a	ND1	61736	58039	94.01%	98.50%	94.77%	54.31%	99.89%
NC3_5b	ND3	48318	45222	93.59%	98.32%	94.26%	54.19%	99.90%
NC3_6a	ND1	56185	52709	93.81%	98.39%	94.52%	54.02%	99.85%
NC3_6b	ND3	68308	64087	93.82%	98.50%	94.78%	54.02%	99.90%
